# Automated Learning of Subcellular Variation among Punctate Protein Patterns and a Generative Model of Their Relation to Microtubules

**DOI:** 10.1371/journal.pcbi.1004614

**Published:** 2015-12-01

**Authors:** Gregory R. Johnson, Jieyue Li, Aabid Shariff, Gustavo K. Rohde, Robert F. Murphy

**Affiliations:** 1 Computational Biology Department, Carnegie Mellon University, Pittsburgh, Pennsylvania, United States of America; 2 Center for Bioimage Informatics, Carnegie Mellon University, Pittsburgh, Pennsylvania, United States of America; 3 Department of Biomedical Engineering, Carnegie Mellon University, Pittsburgh, Pennsylvania, United States of America; 4 Department of Electrical and Computer Engineering, Carnegie Mellon University, Pittsburgh, Pennsylvania, United States of America; 5 Departments of Biological Sciences and Machine Learning, Carnegie Mellon University, Pittsburgh, Pennsylvania, United States of America; 6 Faculty of Biology and Freiburg Institute for Advanced Studies, Albert Ludwig University of Freiburg, Freiburg, Germany; Max Planck Institute for Plant Breeding Research, GERMANY

## Abstract

Characterizing the spatial distribution of proteins directly from microscopy images is a difficult problem with numerous applications in cell biology (e.g. identifying motor-related proteins) and clinical research (e.g. identification of cancer biomarkers). Here we describe the design of a system that provides automated analysis of punctate protein patterns in microscope images, including quantification of their relationships to microtubules. We constructed the system using confocal immunofluorescence microscopy images from the Human Protein Atlas project for 11 punctate proteins in three cultured cell lines. These proteins have previously been characterized as being primarily located in punctate structures, but their images had all been annotated by visual examination as being simply “vesicular”. We were able to show that these patterns could be distinguished from each other with high accuracy, and we were able to assign to one of these subclasses hundreds of proteins whose subcellular localization had not previously been well defined. In addition to providing these novel annotations, we built a generative approach to modeling of punctate distributions that captures the essential characteristics of the distinct patterns. Such models are expected to be valuable for representing and summarizing each pattern and for constructing systems biology simulations of cell behaviors.

## Author Summary

Determining the subcellular location of all proteins is a critical but daunting task for systems biologists, especially when variation between different cell types is considered. Fluorescence microscopy is the main source of information about subcellular location, but large collections of fluorescence images for many proteins are frequently annotated visually and result in assignment only to broad categories. In this paper, we describe automated methods for analyzing images from the Human Protein Atlas to identify nine specific punctate patterns and assign these more specific annotations to 550 proteins many of which previously had little information about subcellular location. We also describe building models of these patterns that will be useful for carrying out systems biology simulations of cellular reactions using accurate spatial distributions.

## Introduction

Fluorescence microscope images can provide important information about the subcellular location of proteins, and automated systems can be used to assign these proteins to major subcellular location classes with accuracy at or above that of human annotators [1, 2]. However, assigning higher resolution annotations to proteins is more difficult, especially for punctate or vesicular patterns. Punctate subcellular localization patterns may arise either from membrane-bound organelles (e.g., transport vesicles) or from macromolecular complexes of sufficient size (e.g., ribonucleoprotein (RNP) bodies), and they may be quite visually similar. We refer to individual components of these patterns collectively as puncta, to encompass both types of structures. These are important for various cellular tasks such as endocytosis, exocytosis and RNA recruitment, storage or degradation. A critical factor for accomplishing many of those tasks is the association of the vesicles or bodies with cytoskeletal components such as microtubules for intracellular transport. Although microtubules are not necessary for short-range transport, they are required for rapid transport of vesicles [3]. The extent to which the distributions of specific puncta are related to that of microtubules remains unclear, as is the extent to which the distributions vary across different cell lines.

Our understanding of cell behavior and the sources of cellular variation can be significantly aided and tested using cell modeling and simulations [4–6]. For this, we need a mechanism to capture the spatiotemporal behavior of cellular substructures, both as a starting point for simulations and to compare against results. Towards this end, we have previously described systems for building image-derived, 2D or 3D generative models of the distributions of either punctate organelles [7, 8] or microtubules [9] within cells. These models are conditional (dependent) on models of cell and nuclear membranes, but they are independent of each other; that is, they do not consider the relationship between puncta and microtubules.

Here we describe a new computational method that allows us to model this relationship. Our method requires images in which both punctate proteins and microtubules are visualized. The Human Protein Atlas (HPA, http://proteinatlas.org) is a rich source of such images, containing high-resolution images of subcellular location patterns for thousands of proteins in several cell lines [10]. To analyze the patterns of punctate proteins in the HPA, we designed a generative model consisting of compact and interpretable features to characterize the population of puncta within a cell, including measurements of microtubule association, relationship to cell geometry, density, intensity and appearance. We have used the features of these models to discover the major modes of variation among punctate patterns, and to assign subclasses of punctate patterns to unannotated proteins.

## Results

### Dependence of protein pattern location on microtubules

**Fig 1:**
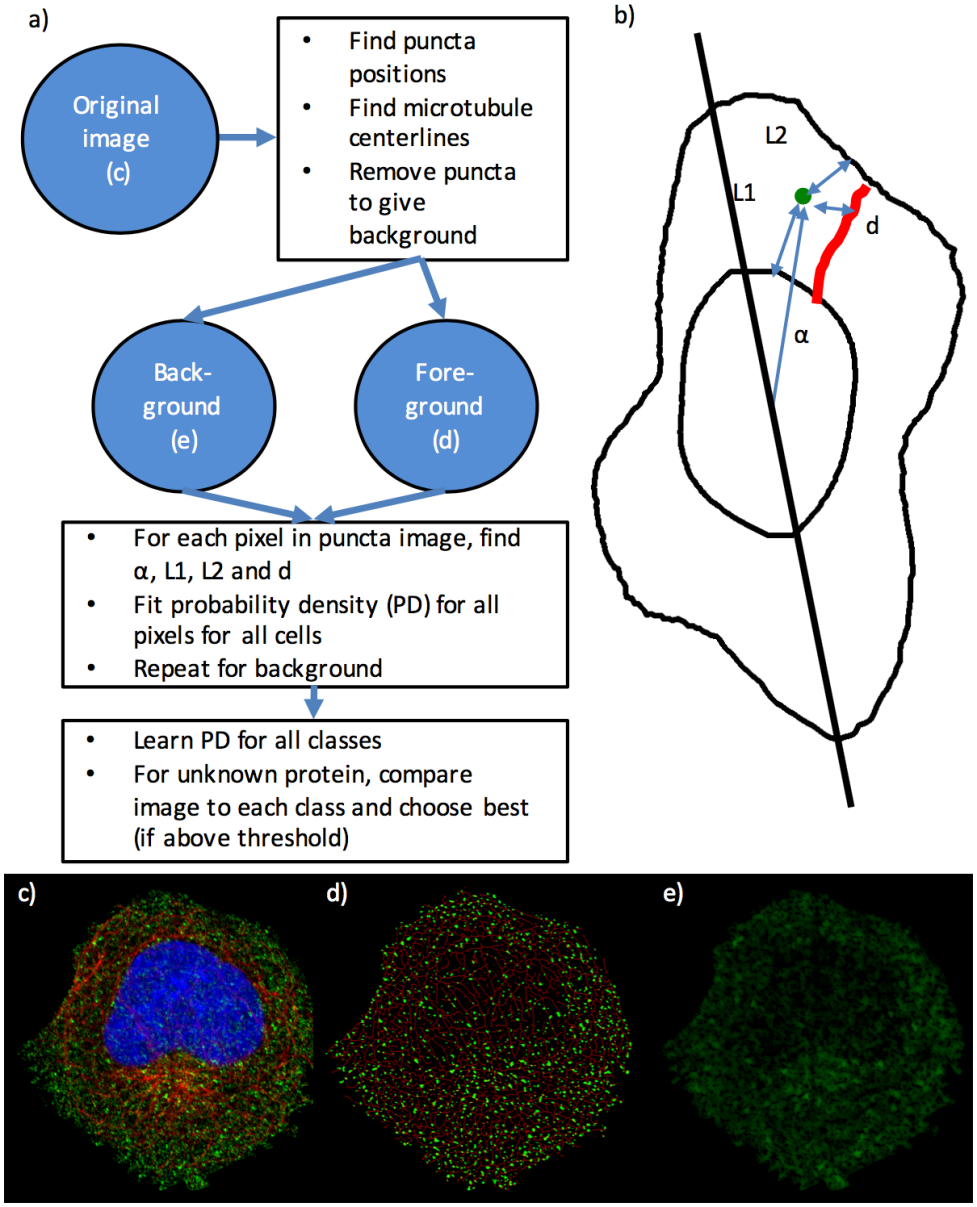
(a) Summary of model learning and classification pipeline. (b) Illustration of coordinate system for probability density function. For each pixel in an image, distance between it and the nearest point on the nuclear membrane (L1) and between it and the nearest point on the cell membrane (L2) are calculated and used to calculate the radial position (*r*) as L1/(L1+L2). In addition, the distance to the nearest point on a segmented microtubule (*d*) and the angle between the pixel and the major axis of the cell (α) are calculated. (c) A two-color image of a vesicular protein (TFRC, transferrin receptor, green) and microtubules (red) in a U-2OS cell. (d) Segmented image of microtubules (red) and puncta (green). (e) Remaining background intensity.

We began by creating an image processing pipeline that identified individual puncta and microtubules in 2D confocal microscopy images from the HPA. As illustrated in Fig 1A, an input image (Fig 1C) is processed to create images of puncta and microtubules (shown as a composite in Fig 1D) and of the remaining background protein fluorescence (Fig 1E). One of our major goals was to generate a model of the distribution of puncta that captures their relationship to microtubules. This would presumably reflect the extent to which puncta were bound to microtubules to accomplish transport to or retention in particular regions of the cell. As a simple measure of this association, we computed the distance (*d*) between each punctum and the nearest microtubule (Fig 1B). We would expect puncta that are bound to microtubules to have a small distance compared to those that are not bound, and perhaps also that the distribution of distances would reflect the extent to which released vesicles diffuse away before being bound again. We added this measure to our previous vesicular object distribution model [8], which included dependence on fractional distance between the nucleus and plasma membrane (*r*, calculated from L1 and L2) and the angle (α) to the major axis of the cell (see Methods). We also created a model for background intensity that was similarly dependent on microtubules and cell shape (see Methods). We combined the estimated parameters from these models with five parameters that describe puncta size and shape and two parameters that measure the amount of fluorescence in puncta and background. This resulted in twenty-two parameters (S1 Table) that can be readily determined from each image of a protein’s subcellular distribution in an individual cell. We used these parameters both as features to describe protein patterns and, later, to construct generative models of punctate patterns.

### Identification of punctate subpatterns and principal modes of variation

**Fig 2:**
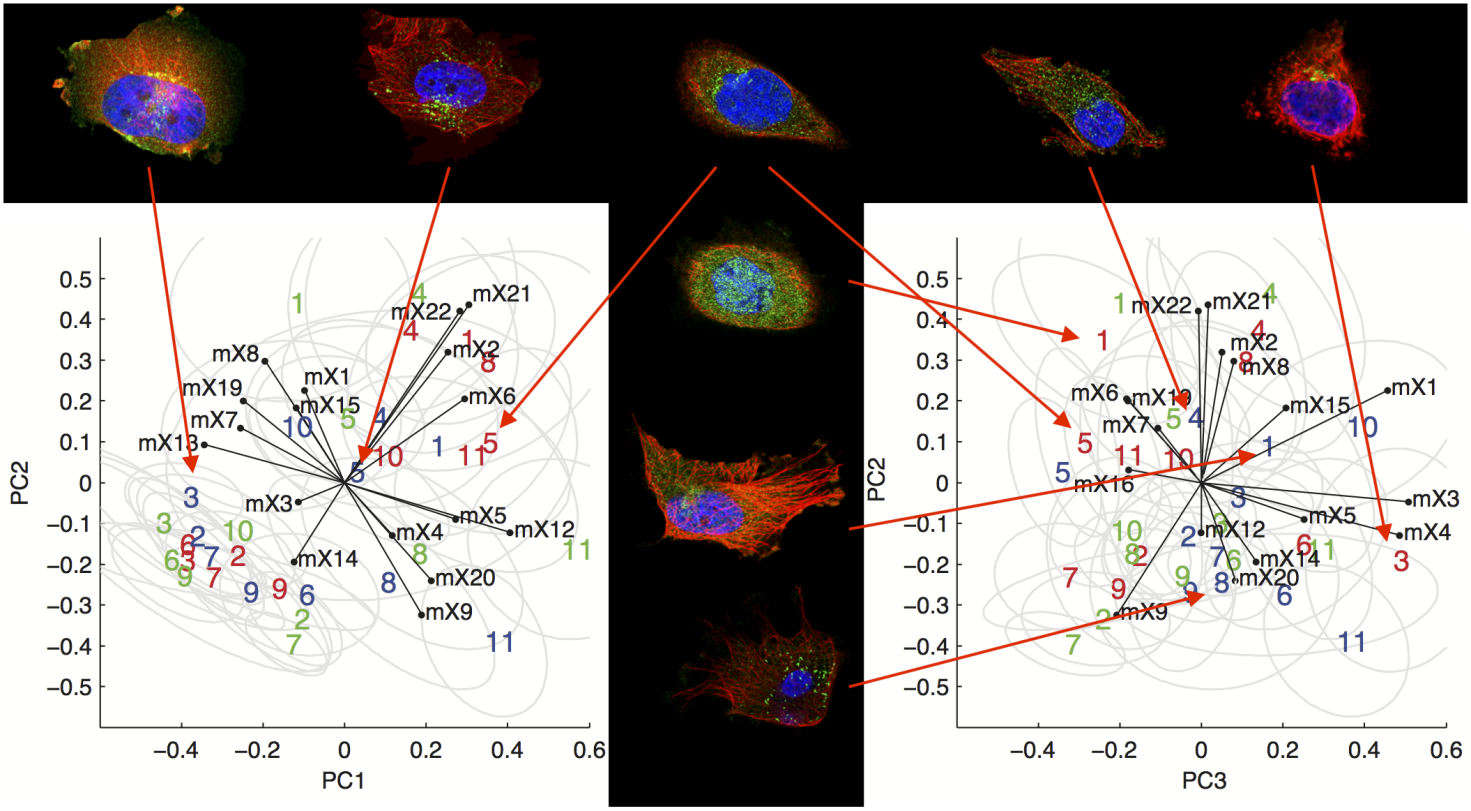
Distribution of cells of the combinations of proteins and cell lines in the first three principal components learned from the whole feature space by PCA. The number for each point indicates the protein index and the color indicates the cell line (red for A-431, green for U-2OS and blue for U-251MG). The gray ellipses represent the scope of 1.5 standard deviations, which contain about 50% to 80% of cells. The arrows summarize the composition of each principal component by showing the direction in which each feature increases (see S1 Table for the list of features). The left panel shows the first and second principal components while the right panel shows the second and third principal components. Feature projections with a magnitude less than 0.1 were removed for visualization purposes.

A number of proteins in the HPA are assigned annotations of “vesicles” or “cytoplasm”. We considered whether we could use HPA images to assign these proteins to a more specific organelle or structure. By examining UniProt annotations and primary literature for proteins whose subcellular location has been reasonably well characterized, we selected eleven proteins that are found in eleven specific types of punctate patterns (Table 1) (we refer to these proteins as “founders” since they enabled us to define specific subtypes). We chose these patterns due to the fact that the proteins showed a similar pattern across all three cell types in the HPA and they represent a wide range of membrane and non-membrane bound compartments (although there are of course additional punctate patterns for which we did not find appropriate founders). In particular, they cover all main compartments of the endomembrane system. We calculated the feature values for all cells for each combination of the eleven proteins and three cell lines. We verified that the features accurately reflect the relationship between vesicles and microtubules by comparing the cumulative distribution of the experimentally measured distance between puncta and microtubules with that calculated from the model; the distributions were very similar for all eleven patterns (S1 Fig). We then asked whether these patterns could be distinguished from each other in HPA images. To provide a visual basis for illustrating how the proteins differed in the features, we calculated the first three principal components. Fig 2 shows the position of each antibody-cell line combination in two projections of this three-dimensional space, as well as representative images along each principal axis. For a given cell line, the eleven patterns are roughly separable, although the position of a given protein sometimes varies from cell line to cell line. For example, proteins 2, 3, 6 and 7 are close together in pc1 and pc2 but separated by pc3. From inspection of the projection of each numerical feature onto the three most significant principal component axes, as well as the example images, it appears that the first component primarily represents variation in features 12, 13 and 5, which capture relationship to microtubules and variation in intensity. The second primarily represents variation in features 21, 22, 2, and 8, which capture intensity and distance from the nucleus, while the third principal component represents variation in features 1, 3, and 4, which capture puncta size and variation in size. This figure does not permit accurate assessment of the overlap between patterns, but is presented to give a visual overview of the major modes of variation with the patterns.

**Table 1:** Proteins used to define punctate subpatterns in this study.

Prot. Num.	Ensembl Gene ID	Gene Name	Gene Description	Structure
1	ENS000000105669	COPE	Coatomer protein complex, subunit epsilon	COPI
2	ENS000000101310	SEC23B	Sec23 homolog B (S. cerevisiae)	COPII
3	ENS000000137312	FLOT1	Flotillin 1	Caveolae
4	ENS000000122705	CLTA	Clathrin, light chain A	Coated Pits
5	ENS000000102189	EEA1	Early endosome antigen 1	Early Endosome
6	ENS000000075785	RAB7A	RAB7A, member RAS oncogene family	Late Endosome
7	ENS000000170088	TMEM192	Transmembrane protein 192	Lysosome
8	ENS000000121691	CAT	Catalase	Peroxisome
9	ENS000000134982	APC	Adenomatous polyposis coli	RNP body
10	ENS000000072274	TFRC	Transferrin receptor	Recycling Endosome
11	ENS000000069329	VPS35	Vacuolar protein sorting 35 homolog (S. cerevisiae)	Retromer

### Constructing a classifier for punctate subpatterns

These results suggest that the feature set may be a reliable basis for measuring variation in punctate patterns, and we therefore sought to determine whether we could use them to predict the compartmental localization of other proteins to one of the eleven patterns. To do this, we first used the features to construct a classification accuracy-derived separability statistic to compare two collections of cells (see Methods) and assessed the extent to which the eleven patterns could be distinguished. We used a classification approach based on Bayes error rate in order to avoid problems with imbalance between the numbers of proteins in each class and to allow for class-specific differences in scale for different features (see Methods).

For each cell type separately, we classified each image as belonging to one of the eleven patterns using hold-out-image cross validation: for each held-out image, we calculated the separability between the cells contained in that image and the cells of each of the founder patterns. The image was given the label of the pattern that was least separable from it. Using this method for each cell type we achieved an average class accuracy of 86.9% (Table 2). We compared these results to those using the same classification procedure but excluding the features relating to microtubule distribution, which resulted in 82.8% average accuracy. This demonstrates that the relationship to microtubules provides information that improves our ability to distinguish punctate patterns. Further examination of Table 2 reveals that the coated pits pattern is the only one that is consistently difficult to distinguish. This may in part be due to the fact that 2D confocal images were used, and thus the features cannot easily distinguish whether puncta are on the surface or inside the cell (for the other surface puncta pattern, caveolae, their distribution or size must allow them to be distinguished).

**Table 2:** Ability to distinguish 11 punctate classes. Classifiers were trained using 5-fold cross validation, and the class of the held out image was predicted. Results are shown for classifiers constructed using all features, and values in parentheses are for training without the microtubule features.

	COPI	COPII	Cav	CP	EE	LE	Lyso	Perox	RNP	RE	Retro
***A-431***											
**COPI**	1 (0.5)	0 (0)	0 (0)	0 (0)	0 (0.5)	0 (0)	0 (0)	0 (0)	0 (0)	0 (0)	0 (0)
**COPII**	0 (0)	0.5 (0.5)	0 (0)	0 (0)	0 (0)	0 (0)	0.5 (0.5)	0 (0)	0 (0)	0 (0)	0 (0)
**Caveolae**	0 (0)	0 (0)	0.5 (0)	0 (0)	0 (0)	0.5 (0)	0 (0)	0 (0)	0 (0.5)	0 (0.5)	0 (0)
**Coated Pits**	0.01 (0)	0.01 (0)	0.01 (0)	0.26 (0.38)	0.14 (0.12)	0.01 (0)	0.01 (0)	0.39 (0.25)	0.01 (0)	0.01 (0)	0.14 (0.25)
**Early Endosomes**	0 (0)	0 (0)	0 (0)	0 (0)	1 (1)	0 (0)	0 (0)	0 (0)	0 (0)	0 (0)	0 (0)
**Late Endosomes**	0 (0)	0 (0)	0 (0)	0 (0)	0 (0)	1 (1)	0 (0)	0 (0)	0 (0)	0 (0)	0 (0)
**Lysosomes**	0 (0)	0.5 (0)	0 (0)	0 (0)	0 (0)	0 (0)	0.5 (1)	0 (0)	0 (0)	0 (0)	0 (0)
**Peroxisomes**	0 (0)	0 (0)	0 (0)	0 (0)	0 (0)	0 (0)	0 (0)	1 (1)	0 (0)	0 (0)	0 (0)
**RNP bodies**	0 (0)	0 (0)	0 (0)	0 (0)	0 (0)	0 (0)	0 (0)	0 (0)	1 (1)	0 (0)	0 (0)
**Recycling Endosomes**	0.17 (0.33)	0 (0)	0 (0)	0 (0)	0.17 (0)	0 (0)	0 (0)	0 (0)	0 (0)	0.67 (0.67)	0 (0)
**Retromer**	0 (0)	0 (0)	0 (0)	0 (0)	0 (0)	0 (0)	0 (0)	0 (0)	0 (0)	0.5 (0)	0.5 (1)
***U-2OS***											
**COPI**	1 (1)	0 (0)	0 (0)	0 (0)	0 (0)	0 (0)	0 (0)	0 (0)	0 (0)	0 (0)	0 (0)
**COPII**	0 (0)	1 (1)	0 (0)	0 (0)	0 (0)	0 (0)	0 (0)	0 (0)	0 (0)	0 (0)	0 (0)
**Caveolae**	0 (0)	0 (0)	1 (1)	0 (0)	0 (0)	0 (0)	0 (0)	0 (0)	0 (0)	0 (0)	0 (0)
**Coated Pits**	0 (0)	0 (0)	0 (0)	0.43 (0.57)	0 (0)	0 (0)	0 (0)	0 (0)	0 (0)	0 (0)	0.57 (0.43)
**Early Endosomes**	0 (0)	0 (0)	0 (0)	0 (0)	1 (1)	0 (0)	0 (0)	0 (0)	0 (0)	0 (0)	0 (0)
**Late Endosomes**	0 (0)	0 (0)	0 (0.5)	0 (0)	0 (0)	1 (0.5)	0 (0)	0 (0)	0 (0)	0 (0)	0 (0)
**Lysosomes**	0 (0)	0 (0)	0 (0)	0 (0)	0 (0)	0 (0)	1 (1)	0 (0)	0 (0)	0 (0)	0 (0)
**Peroxisomes**	0 (0)	0 (0)	0 (0)	0 (0)	0 (0)	0 (0)	0 (0)	0.89 (0.78)	0 (0)	0 (0)	0.11 (0.22)
**RNP bodies**	0 (0)	0 (0)	0 (0)	0 (0)	0 (0)	0 (0)	0 (0)	0 (0)	1 (1)	0 (0)	0 (0)
**Recycling Endosomes**	0 (0)	0 (0)	0 (0)	0 (0)	0 (0)	0 (0)	0 (0)	0 (0)	0 (0)	1 (1)	0 (0)
**Retromer**	0 (0)	0 (0)	0 (0)	0 (0)	0 (0)	0 (0)	0 (0)	0 (0)	0 (0)	0 (0)	1 (1)
***U-251 MG***											
**COPI**	1 (1)	0 (0)	0 (0)	0 (0)	0 (0)	0 (0)	0 (0)	0 (0)	0 (0)	0 (0)	0 (0)
**COPII**	0 (0)	1 (0.5)	0 (0)	0 (0)	0 (0)	0 (0)	0 (0)	0 (0)	0 (0.5)	0 (0)	0 (0)
**Caveolae**	0 (0)	0 (0)	1 (1)	0 (0)	0 (0)	0 (0)	0 (0)	0 (0)	0 (0)	0 (0)	0 (0)
**Coated Pits**	0 (0)	0 (0)	0 (0)	0.67 (1)	0 (0)	0 (0)	0 (0)	0 (0)	0 (0)	0 (0)	0.33 (0)
**Early Endosomes**	0 (0)	0 (0)	0 (0)	0 (0)	1 (1)	0 (0)	0 (0)	0 (0)	0 (0)	0 (0)	0 (0)
**Late Endosomes**	0 (0)	0 (0)	0 (0)	0 (0)	0 (0)	1 (1)	0 (0)	0 (0)	0 (0)	0 (0)	0 (0)
**Lysosomes**	0 (0)	0 (0)	0 (0)	0 (0)	0 (0)	0 (0)	1 (1)	0 (0)	0 (0)	0 (0)	0 (0)
**Peroxisomes**	0.08 (0.15)	0 (0)	0 (0)	0 (0)	0 (0)	0 (0)	0 (0)	0.77 (0.62)	0 (0)	0.08 (0.15)	0.08 (0.08)
**RNP bodies**	0 (0)	0 (0.5)	0 (0)	0 (0)	0 (0)	0 (0)	0 (0)	0 (0)	1 (0.5)	0 (0)	0 (0)
**Recycling Endosomes**	0 (0.17)	0 (0)	0 (0)	0 (0)	0 (0)	0 (0)	0 (0)	0 (0)	0 (0)	1 (0.83)	0 (0)
**Retromer**	0 (0)	0 (0)	0 (0)	0 (0)	0 (0)	0 (0)	0 (0)	0 (0)	0 (0)	0 (0)	1 (1)

### Annotation of other punctate proteins

We next asked whether the classification approach could be used to assign a punctate subpattern annotation to an image of proteins other than the founders. We did not want to simply assign the subcellular location of the class that a protein was most similar to (since the protein might not actually be from any of our classes), but wanted to ensure that we only assigned annotations for proteins with a high degree of similarity to one of the founders. For each cell type, we determined a threshold on the separability statistic that could be used to determine whether or not a new protein should be assigned to a particular class. This threshold was determined as the optimal point of the receiver operating characteristic curve (see Methods and S2 Fig) for each cell type.

To assign subcellular location to a new image, we measured the separability between it and each founder pattern. If the value for one of the patterns was below the threshold, we assigned the corresponding pattern label to that image. In the rare case of an image being below the threshold of multiple patterns, we assigned it the label “ambiguous.” This classification procedure was applied to the remainder of images in the HPA dataset; the results are contained in S1 Dataset. One hundred and twenty-five proteins were identified as belonging to one of the eleven classes in A-431, 60 in U-2OS, and 365 in U-251 MG. The list of the most confident assignments is shown in Table 3. With the goal of providing improved annotations for protein databases, we also generated an XML file that can be used to update those databases. The file (S2 Dataset) contains information on HPA antibody IDs, gene targets and proposed annotation. Due to the nature of immunofluorescence tagging, a sequence-specific tag may be present on more than one protein isoform, each of which may show a condition-specific localization pattern. With that in mind, we also report the known protein gene products provided by ENSEMBL 79, and the percentage of matching peptides after alignment between the gene-product and antigen sequences in the region spanned by the antibody. We also provide annotations to all protein isoforms that match the antibody sequence. For those proteins and isoforms that have a high confidence location assignment, we also provide an XML file for updating their UniProt record (S3 Dataset).

**Table 3:** Top-ranked proteins assigned to one of the ten high-confidence subpatterns. The top protein for each cell type for each subpattern (except Coated Pits) is included if its separability is less than 0.70 (which is more selective than the threshold determined in S2 Fig). The separability measures for all proteins are included in S1 Dataset.

Antibody ID	EMBL Gene ID*	Gene Name	Gene Description	Proposed Annotation
*A-431*				
HPA017909	172113	NME6	NME/NM23 nucleoside diphosphate kinase 6	COPII
HPA007722	073417	PDE8A	Phosphodiesterase 8A	Caveolae
HPA038052	110013	SIAE	Sialic acid acetylesterase	Early Endosomes
HPA015055	141867	BRD4	Bromodomain containing 4	Lysosomes
HPA007875	149968	MMP3	Matrix metallopeptidase 3	RNP bodies
HPA029806	196305	IARS	Isoleucyl-tRNA synthetase	Retromer
*U-2OS*				
HPA003220	204920	ZNF155	Zinc finger protein 155	COPII
HPA003607	100665	SERPINA4	Serpin peptidase inhibitor, clade A (alpha-1 antiproteinase, antitrypsin), member 4	Caveolae
HPA004167	108774	RAB5C	Member RAS oncogene family	Early Endosomes
HPA003280	167085	PHB	Prohibitin	Late Endosomes
HPA014907	162366	PDZK1IP1	PDZK1 interacting protein 1	Lysosomes
HPA002883	141665	FBXO15	F-box protein 15	RNP bodies
HPA010570	163840	DTX3L	Deltex 3-like (Drosophila)	Recycling Endosomes
HPA041566	180096	SEPT1	Septin 1	Retromer
*U-251 MG*				
HPA023476	147174	ACRC	Acidic repeat containing	COPI
HPA003084	139915	MDGA2	MAM domain containing glycosylphosphatidylinositol anchor 2	COPII
HPA017770	160886	LY6K	Lymphocyte antigen 6 complex, locus K	Caveolae
HPA002946	004961	HCCS	Holocytochrome c synthase	Late Endosomes
HPA003524	103811	CTSH	Cathepsin H	Lysosomes
HPA015313	103034	NDRG4	NDRG family member 4	RNP bodies

*all begin with ENSG00000

In order to provide an independent assessment of the accuracy of the annotation procedure, we searched for literature describing the localization of the most confident annotations. We were able to find literature supporting our proposed labeling for many of the proteins (although they had often only been analyzed in other cell types). For example, of the top hits for A-431 cells, BRD4, has been suggested to be involved in the lysosome protolytic pathway [11]. For U-2OS, top hit RAB5C is a classic early endosomal protein [12], and prohibitin (PHB) is a multifunctional membrane protein [13] one of whose roles is in regulation of degradation of PAR1 [14]. For U-251MG cells, the top hits include cathepsin H (CTSH), a lysosomal enzyme, DTX3L, which regulates endosomal sorting [15], and LY6K, which, like other Ly6 antigens, is associated with glycosylphosphatidyl inositol-anchored glycoproteins (such as TEX101 [16]) that are typically found in caveolae. These findings increase our confidence in the proposed annotations.

Many of the proteins analyzed (which were all proteins assigned “vesicles” or “cytoplasm” annotations) were not assigned with high confidence to any of the 11 patterns. There are at least three potential reasons for this. First, the staining may be of low enough intensity or quality that foreground cannot be adequately identified. Second, the unassigned proteins may be cytoplasmic proteins without a discernible punctate pattern, or vesicular proteins from an organelle that we have not considered. Third, they may be present in more than one of the eleven patterns, such that their pattern does not match well enough to any of them.

### Comparison of models for different patterns

Our models allow us to ask whether different punctate subclasses differ in their relationship to microtubules. We performed a simple characterization of this relationship by calculating the average actual distance of each punctum from microtubules, as well as the average distance from microtubules predicted by our fitted model. S3 Fig shows a comparison of these two distances for each pattern across all cell types and for each combination of pattern and cell type. A confidence interval on the average distance from microtubules was determined via the Tukey-Kramer method after two-way ANOVA [17] (across proteins and cell types). All of the symbols are quite near the diagonal, indicating that the model is in high agreement with the measurements. When averaged across all three cell types, retromer, recycling endosomes, and early endosomes show the closest association with microtubules, and RNP bodies, COPI vesicles and coated pits show the least. When each combination of protein and cell type is considered separately, we see greater variability in the distances (perhaps due to differences in microtubule-binding proteins or cell size or shape). COPII, lysosomes and COPI show the least variation across the three cell types, and coated pits and recycling endosomes show the greatest.

**Fig 3:**
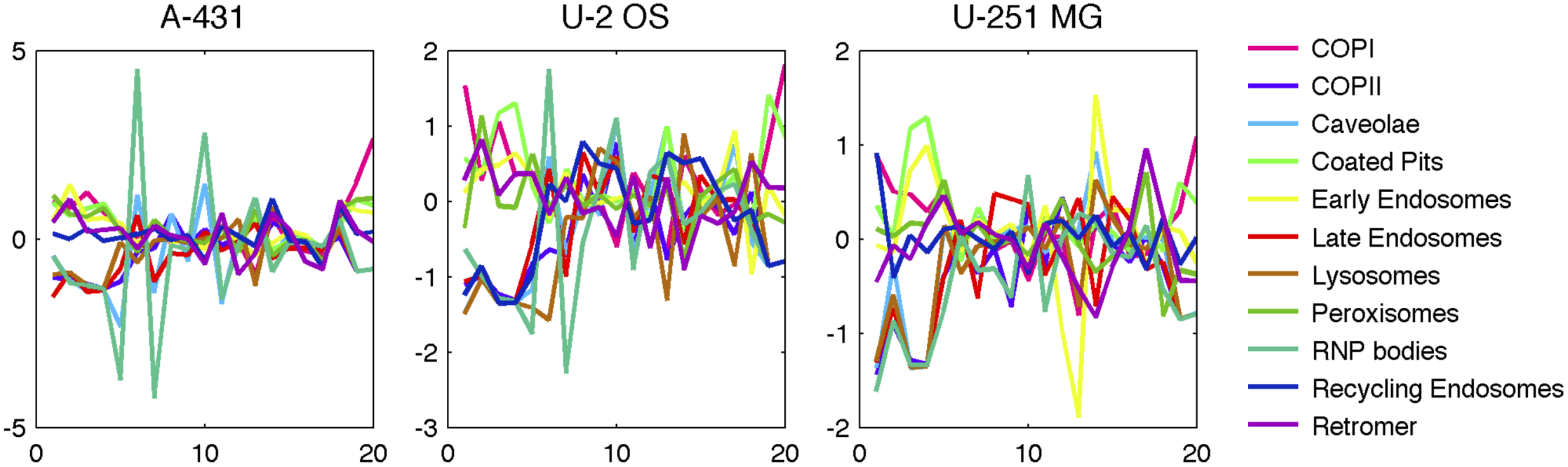
Comparison of model features for different patterns. The values for each feature were z-scored to put them on the same scale across features, and the average value for each feature is shown as a function of the feature number (see S1 Table for feature definitions).

Another way in which we can compare the different patterns is by examining the differences in the model features among them. A simple visualization of this is shown in Fig 3, in which the relative values of each feature are shown for each pattern. In U-2OS, for example, the first four features (relating to size and intensity) clearly distinguish the group of RNP bodies, late endosomes, recycling endosomes, lysosomes and COPII from the others, and a high value for mx5 (number of puncta) separates RNP bodies from this group. Other distinguishing features or feature combinations can also be identified, such as retromers having the lowest value for mx12 (consistent with their close association with microtubules). These differences provides a interpretable rationale for the ability of the classifiers to distinguish the patterns.

### Generative model of punctate protein distributions

A difficult question that frequently gives rise to controversy is how to best describe the subcellular pattern of a given organelle or structure (especially a novel one). Descriptions using unstructured text or Genome Ontology terms defer the question by assuming that the words will be sufficient for the reader to be able to mentally construct the pattern. An alternative is to show an example image, but this does not give an idea of the variation in the pattern (one can find differences between any two example images, but this does not address whether those differences are statistically significant). Unfortunately these two methods of conveying information about the distribution and variation in protein pattern do not provide a quantitative, or much less a probabilistic or statistical representation of the observed pattern. Alternatively, one can give values for a descriptive feature vector or matrix for each pattern (which can be used for a classifier) but this allows one only to recognize new examples but not to produce an example of the pattern. Feature vectors also do not necessarily allow an explicit model of the relationship between cell components. Of course, none of the approaches above are helpful if we desire an *in silico* representation of the cell geometry and expressed patterns (i.e., the consumer of the representation is a computer rather than a cell biologist). For example, information about subcellular patterns is needed for accurate mathematical simulations of cell biochemistry and behavior [4–6]. As a solution, we have introduced the building of generative models of cell organization directly from images [7, 8, 18–20]. The intent is for these models to capture the underlying properties of a particular pattern; in statistical terms, to capture the distribution from which all examples of that pattern are drawn. Such a model can be used to synthesize new cell images from that distribution.

**Fig 4:**
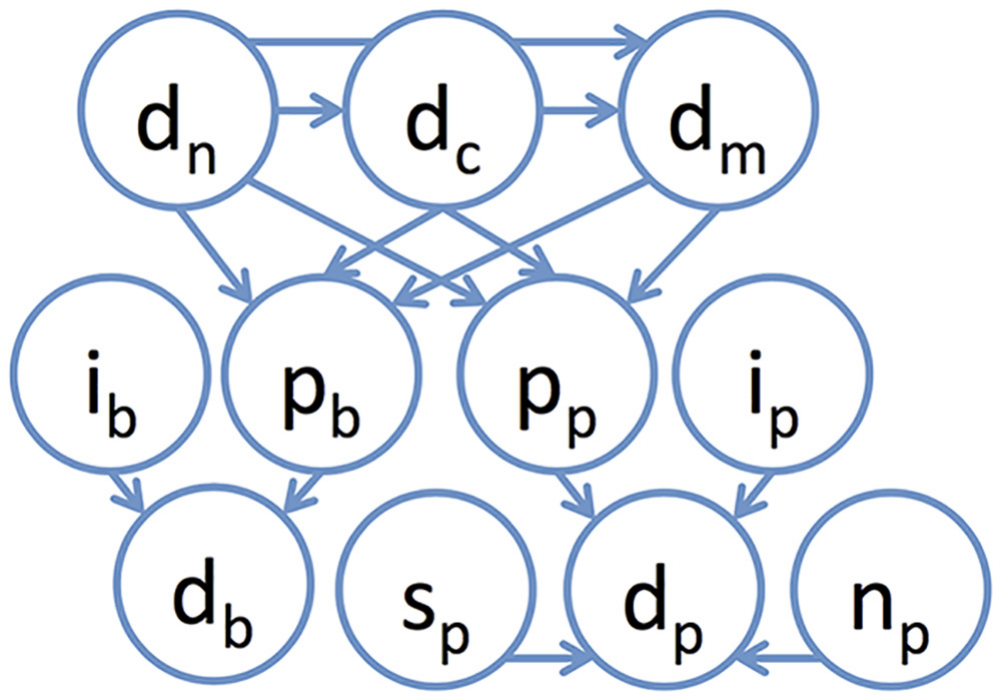
Graphical model representation for the Bayesian hierarchical framework of generative model of puncta conditioned on cell geometry and microtubules. A nuclear shape is drawn from d_n_, a cell shape is drawn from d_c_, dependent on the nuclear shape [8]. A microtubule pattern is synthesized from d_m_ dependent on the generated cell and nuclear shape [9]. The distribution of shape and positions of puncta, d_p_, is modeled with components p_p_, which models the position of puncta dependent on the cell, nucleus and microtubule pattern, and n_p_, s_p_ and i_p_, which independently model the number, size and intensity of puncta. The background pattern is similarly generated dependent on the cell, nucleus and microtubule pattern with p_b_, and its intensity is determined with i_b_.

**Fig 5:**
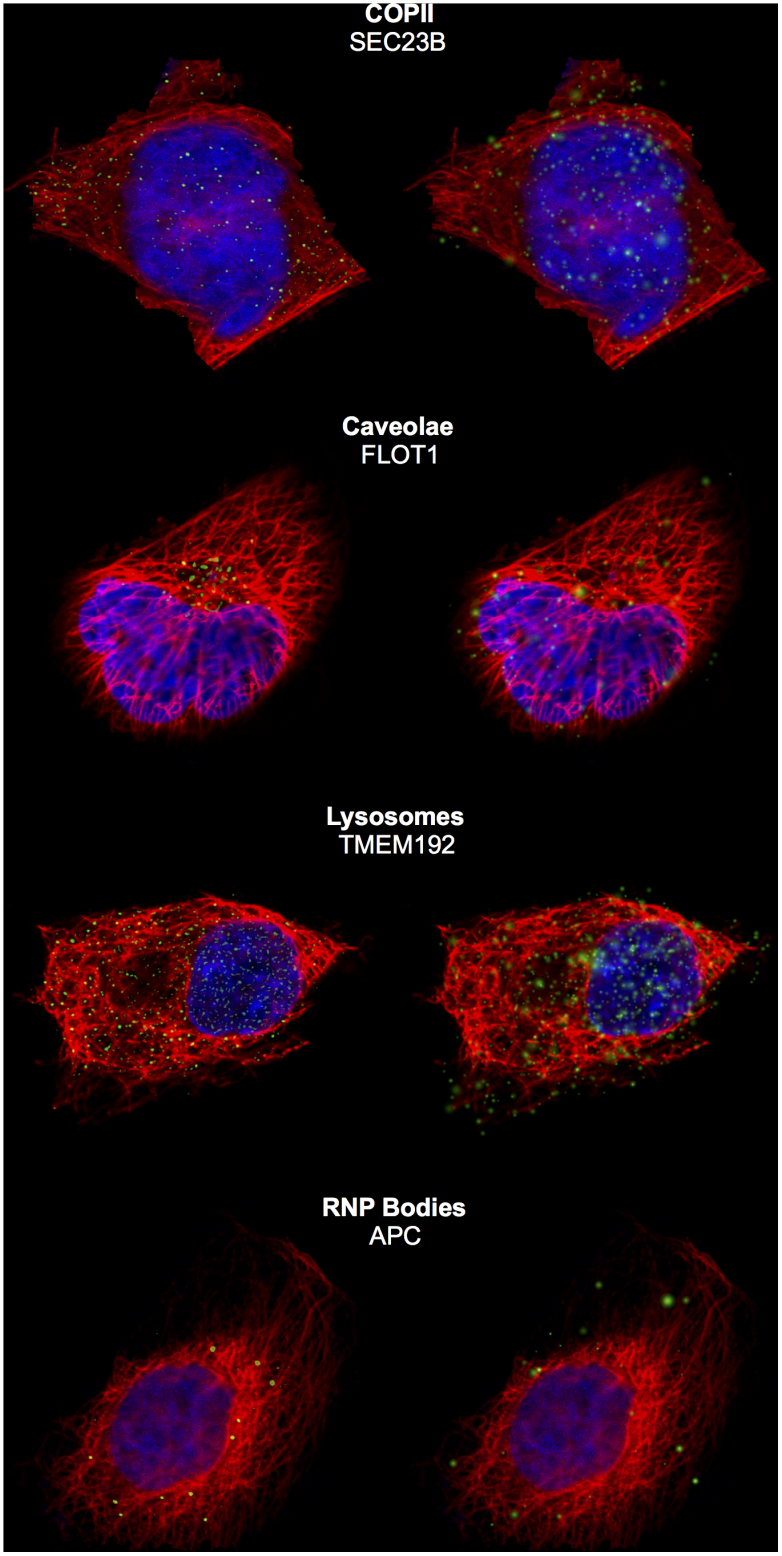
Representative images from four patterns and corresponding synthesized images in U-2OS cells. The left column shows cell images closest to the median of parameter space for cells of that pattern, and the right column shows synthesized cells from the generative model of protein pattern conditional on cell geometry and microtubules of the left panel. The green, red and blue channels represent puncta, microtubules and nuclei, respectively.

We therefore constructed a generative model of punctate patterns whose structure is shown in Fig 4. The model starts with models of nuclear and cell shape (d_n_, d_c_) and microtubule distribution (d_m_) and links them to models of puncta distribution using mx7 through mx11 to capture dependence on cell shape and mx12 and mx13 to capture dependence on microtubules (see Methods). Additionally the size, shape and intensity of vesicles are modeled independently of the cell shape and microtubules with mx1 through mx6. The background intensity is similarly modeled dependent on cell shape and microtubules (mx14 through 20) and scaled to match the fraction of intensity with mx21 and mx22. We illustrate that the images generated from the models learned for each of the pattern classes are similar to real images in Fig 5 and S4 Fig.

**Fig 6:**
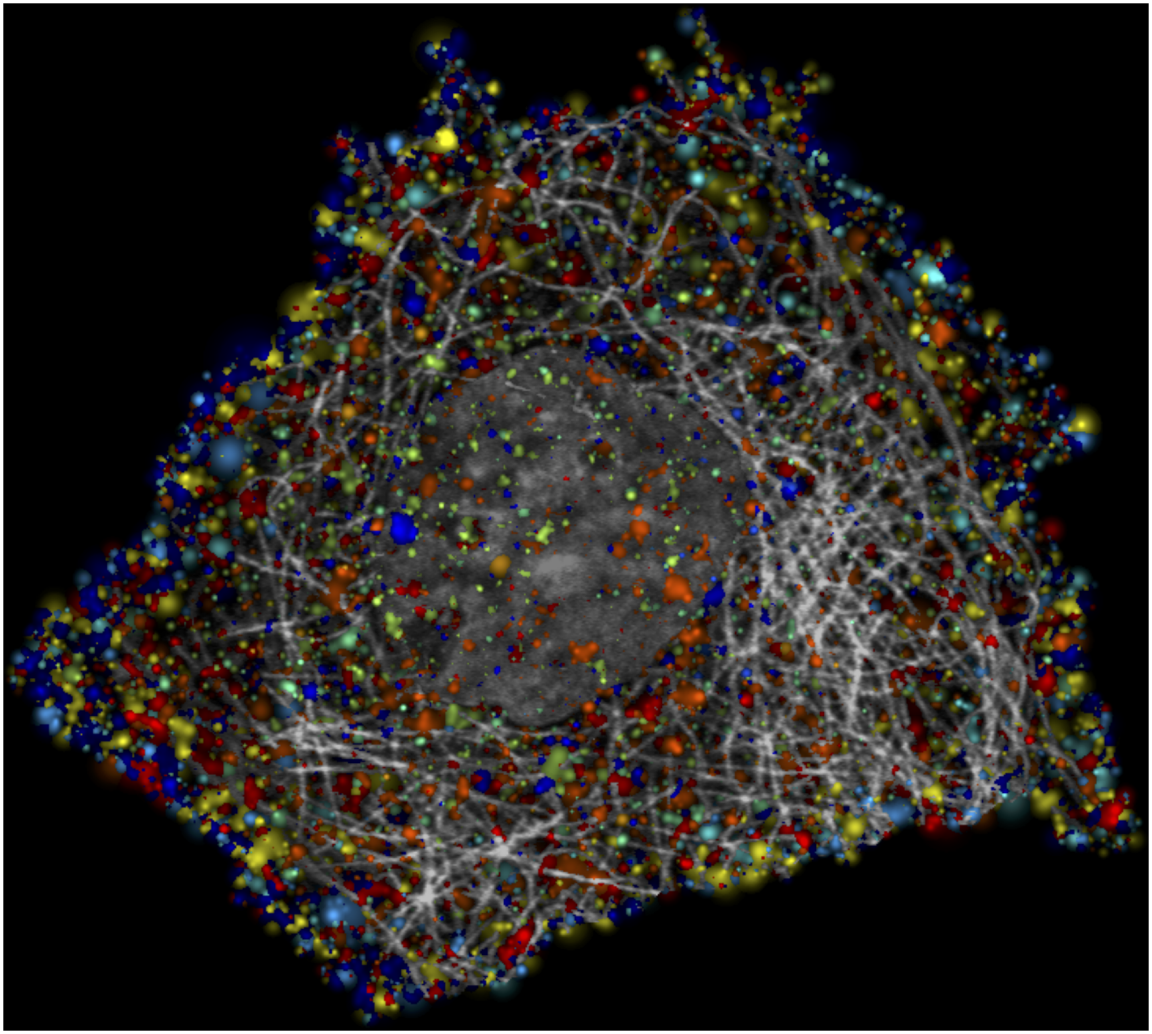
Synthetic cell image containing eleven punctate patterns. Synthetic distributions for all patterns were independently created in the same cell; this assumes that positions of puncta do not affect each other (e.g., that peroxisomes are not more or less likely to be near RNP bodies). The nucleus is shown in dark grey and microtubules in light gray. Colors for patterns are the same as in Fig 3.

Assuming that the distributions of the eleven punctate patterns are independent of each other, we can combine the models and synthesize cells containing all eleven. Fig 6 shows an example of a “typical” cell under this assumption (using the average values of all model parameters).

## Discussion

With the development of systems to fluorescently tag and acquire images of thousands of subcellular protein patterns, a need arose for automated methods to analyze and model the patterns in these images [21]. The goals of such analyses include, but are not limited to, determining the organelles to which different proteins localize and studying the statistical dependency between different protein patterns. However, previous methods have not been able to recognize subpatterns of the major organelle types. Furthermore methods are needed to describe the relationships between cellular components in a way that is not only human-interpretable, but allows us to generate new examples of these patterns for future use in cell simulations [22].

Here we have described a new framework to build models of subcellular punctate patterns conditional on cell geometry and microtubules. These models use interpretable features that capture specific ways in which punctate subpatterns differ between cell types (such as the differences noted at the beginning of the Results) and can generate synthetic cell instances representative of the modeled population. We demonstrated the value of this framework by learning models directly from images of eleven well-characterized punctate protein patterns in three cell types. We showed that the major variation in these patterns corresponded to dependence on microtubules, total intensity, and puncta size and shape. Given the model parameters we constructed a pipeline demonstrating both the high discriminative ability of this model across patterns of the same cell type and the ability to automatically assign annotations to 550 proteins (many of which had been poorly characterized previously with respect to subcellular location).

High-content screening and analysis have become increasingly frequent, including subtle analysis of location changes induced by chemical compounds or inhibitory RNAs and proteome-scale analysis of patterns. The features we have described should be useful for refining the ability to distinguish different vesicular and punctate patterns, and, most importantly, to provide an interpretable and portable basis for comparing them.

The work presented here represents an important step towards bridging detailed models learned from large collections of images for proteins contained in discrete objects with models of microtubule network growth learned by inverse modeling [9, 18]. It serves as an important component of our CellOrganizer project (http://cellorganizer.org/) [20], which aims at capturing a detailed model of the spatial organization and relationships between different subcellular location patterns. We plan to extend this work by merging it with models of subcellular pattern dynamics, as well as extend the model to capture further dependency between components. It is hoped that approaches like this will enable the construction of models that capture essential cell behaviors without requiring the simultaneous measurement of the thousands of different proteins in the same living cell, something that is infeasible with current technology.

## Materials and Methods

### Image collections

The data used here were confocal immunofluorescence microscopy images of fixed cells from A-431, U-2OS and U-251MG cell lines from HPA [10]. All antibodies whose subcellular pattern was annotated as “vesicles” or “cytoplasm” were chosen (a total of 2357, 3038, and 1730 proteins for each line; S1 Dataset contains the complete list of proteins analyzed). The images were analyzed as 8-bit TIFF images with three channels each obtained using a different emission wavelength of fluorescence from a single image field. The three channels show the locations of a specific punctate protein, a nuclear stain, and microtubules. Each of the images is 1728 × 1728 pixels and the pixel size corresponds to 0.08 microns in the sample plane. Founder proteins for eleven patterns were chosen as described in the Results. After segmenting the image fields for these proteins into single cell regions using a seeded watershed method [2], the set of founder images was found to contain 1099 cells, 333 from A-431, 327 from U-2OS and 439 from U-251MG (the number of cells for each of the 33 *combinations* of antibody and cell line varied from 12 to 85).

### Parameterization of microtubules and puncta

In cell images, due to variation in fluorescence intensity in the cytoplasm, segmentation of puncta and microtubules from protein pattern images poses a difficult problem where global threshold-based methods may over-threshold regions of the cytoplasm containing low-intensity structures. The input cell image was de-noised by blurring with a Gaussian filter with standard deviation of 0.75. We isolated high spatial-frequency foreground and low spatial-frequency background intensity images by low pass filtering the smoothed image with a Gaussian filter of 4-pixel standard deviation, and subtracted this background image from the smoothed image, resulting in an image of high-frequency foreground signal (i.e. puncta). The negative-valued pixels of the foreground signal were removed, and the foreground image was subtracted from the first smoothed image, to get the background image (both of which sum to the total image intensity). To increase the speed at which a Gaussian mixture model could be fit over the foreground image, we excluded all pixels below the Ridler-Calvard threshold [23] and all single-pixel objects. We used the skeletonized foreground signal of the microtubule image to model the distances of objects from microtubules. This approach resulted in reasonable definition of both puncta and microtubules and was sufficient to capture variation across the founder patterns analyzed in this paper.

### Computing the distance between each punctum and the nearest microtubule

The centroids of all puncta were computed by fitting a mixture of Gaussians to distinguish overlapping puncta [7]. The distance between the centroid of each punctum and its nearest microtubule was found using a distance transform of the skeletonized microtubule image.

### Capturing vesicle and background position relative to microtubules and cell and nuclear boundaries

A probability density function (PDF) for the position of puncta (p_p_) relative to the cell geometry and microtubules was estimated by extending the model previously described [8] by adding a terms describing the distance from microtubules, *d*:

$$P(r,a,d)\;=\;\frac{e^{\beta_{0}+\beta_{1}r+\beta_{2}r^{2}+\beta_{3}sin\alpha +\beta_{4}cos\alpha +\beta_{5}d+\beta_{6}d^{2}}}{1+e^{\beta_{0}+\beta_{1}r+\beta_{2}r^{2}+\beta_{3}sin\alpha +\beta_{4}cos\alpha +\beta_{5}d+\beta_{6}d^{2}}}$$ (1)

The terms *β*_1_ through *β*_4_ describe the dependency of objects on radial and angular coordinates in relation to the shape of the cell [2, 8], and *β*_5_ and *β*_6_ describe the dependency of objects to be localized in relation to the microtubules. We similarly constructed a PDF for the background intensity (which presumably results from soluble, non-punctate protein).

### Generative models

The Bayesian hierarchical framework for the generative model for puncta is shown in Fig 3 as a graphical model. A multivariate statistical model was constructed from the independent distributions of values of the following statistics from each cell: puncta size (s_p_), puncta per cell (n_p_), and intensity (i_p_).

Synthetic cell instances were created starting from the cell and nuclear boundaries and microtubule image of a randomly-selected cell. (They can also be created by first generating cell and nuclear boundaries and microtubule distributions using models learned previously for the three cell lines [18].) To add puncta to a cell, values were sampled for the number of puncta per cell (n_p_) and the size (s_p_) and fluorescence intensities (i_p_)) for each punctum from distributions learned from 2D HPA data. These were used to generate puncta using the Gaussian object based generative model [8]. Positions for them were sampled from the vesicle position PDF from the model above after morphing to the specific cell geometry. Background fluorescence was added using the learned PDF from the background images, scaled to match a draw from the total background intensity distribution learned from images.

### Image classification

The assignment of subcellular annotations to images of cells is a classification task with complications found in many biological contexts; specifically being the structured nature of data (cells with the same antibody should all be assigned the same label), the inseparability of class data (proteins with different biochemical properties may have similar localization patterns), and imbalanced number of observations(some images may contain many cells while others have few). We designed a classification method to specifically address the above complications.

Given pattern parameterizations corresponding to cells of two collections (all cells contained in two images), we perform a balanced classification task to determine how distinguishable the two collections are. For each pair of images, we hold out a subset of cells and train an SVM by weighting the training data such that there is a uniform prior across the classes. We then classify the hold-out and count the frequency at which the hold-out was assigned the correct collection, approximating the Bayes Error rate [24]. This approach is similar to other methods used in genomics [25]. We take the average classification accuracy across all cell classification tasks (whether or not the cells belonging to the two images are assigned the same subcellular pattern) as a measure of how distinguishable the two collections are, resulting in a possible range of values from 1 (totally separable) to 0 (completely inseparable). In virtually all cases, the measure of difference lies between 0.5 and 1. We will refer to this measure as “dissimilarity”.

To determine a threshold on dissimilarity, at which we can say two collections belong to the same or different patterns, the pipeline treats images of each of our basis patterns as their own collection (with multiple images of each pattern) and performs the above classification task using cells contained in each image. An ROC curve is constructed, indicating the true and false positive classification rates as a function of increasing dissimilarity. For each cell type we constructed an upper-bound of dissimilarity (above which is considered “not the same annotation”) by the cutoff determined at the location where the upper-left-most point of the ROC curve intersects with a slope of $\frac{TN+FP}{TP+FN}$, where TN, FP, TP and FN are the counts of true negative, false positive, true positive and false negatives respectively. When comparing our basis set to images containing cells of unknown protein localization, we assign the unknown pattern the label of any basis pattern that is within the similarity threshold. These thresholds were 0.78846, 0.70588 and 0.72093 for A-431, U-2OS, and U-251MG, respectively.

### Software availability

All software and data used for this work is available as a reproducible research archive (http://murphylab.web.cmu.edu/software). The software will also be available as part of the open source CellOrganizer system (http://CellOrganizer.org). The segmentation and feature calculation pipeline can be used separately.

## Supporting Information

S1 Fig Quality of fitted distributions for punctate proteins. P-P plots comparing the CDFs of the probability of vesicle given distance from microtubule for the fitted model and the empirical distribution are shown for the median cell of each pattern (the same cells as shown in Fig 5 and S4 Fig).
(TIF)

S2 Fig Determination of annotation threshold. Receiver operating characteristic curves for the accuracy statistic for determining the in-class threshold are shown for the three cell types. The accuracy corresponding to the optimal threshold is shown as a black circle (see Methods).
(TIF)

S3 Fig Comparison of average distance of puncta from microtubules measured empirically and in our fitted model across proteins, cell types, and proteins and cell types. Each symbol represents a cell type; square for A-431, diamond for U-2 OS and circle for U-251 MG. The lines represent confidence intervals using Tukey’s range test for the empirical data (x-axis) and fitted model (y-axis) after 2-way ANOVA.
(TIF)

S4 Fig Representative images from seven patterns and corresponding synthesized protein pattern in U-2OS cells. The left column shows cell images closest to the median of parameter space for cells of that pattern, and the right column shows synthesized protein patterns from the generative model of protein pattern conditional on cell geometry and microtubules of the left panel.
(TIF)

S1 Dataset Results for comparison of HPA proteins to the eleven punctate subpattern classes. The values in the columns for each subpattern are the separability measures for all cells of a given protein with the cells of the founder protein(s) for that subpattern.
(XLS)

S2 Dataset Updated protein annotations resulting from this work. The file is in XML format appropriate for incorporation into protein databases. These entries are only for those proteins assigned to a single pattern using the thresholds determined in S2 Fig.
(XML)

S3 Dataset Updated protein annotations for the UniProt database. The information in S2 Dataset is reformatted and includes Genome Ontology terms to be assigned to each protein.
(XML)

S1 Table Generative model parameters. Radial position is defined as *r* = *L*1/(*L*1+*L*2) where *L*1 is the distance between the center of each punctum and the nuclear membrane, and *L*2 is the distance from the center of each punctum to the cell membrane. Therefore, *r* is positive if the punctum is outside of the nucleus and negative inside. α is the angle between the major axis of the cell and the vector from the center of cell to the center of a punctum. The generative model component that a given feature is used for is also shown (see Fig 3).
(DOCX)
