# Supplementary figures and images for: Automated Learning of Subcellular Variation among Punctate Protein Patterns and a Generative Model of Their Relation to Microtubules
Source: PLoS Comput Biol. 2015 Dec 1;11(12):e1004614. doi: 10.1371/journal.pcbi.1004614 (PMC4704559; doi:10.1371/journal.pcbi.1004614)

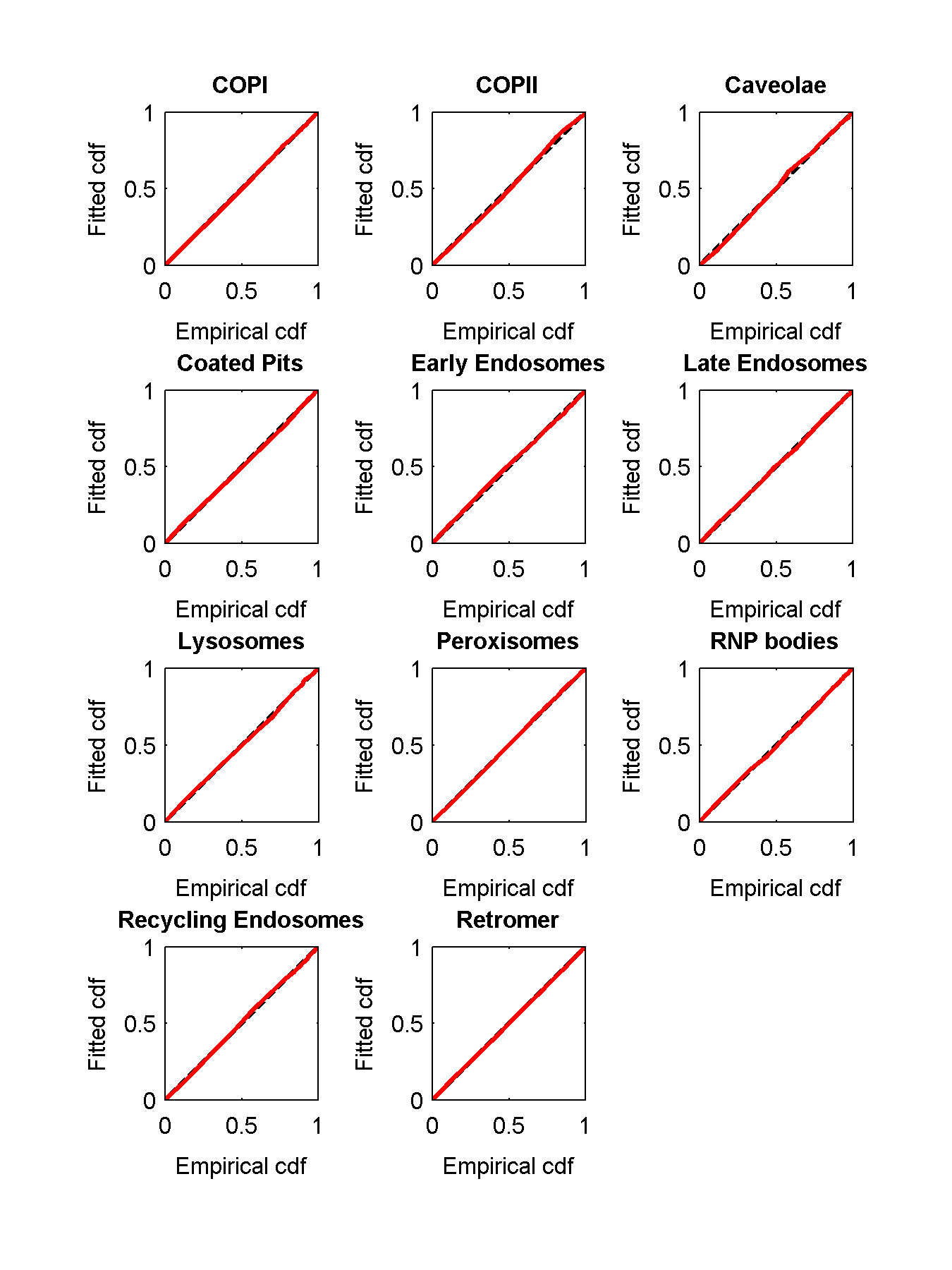

Supplement: S1 Fig — P-P plots comparing the CDFs of the probability of vesicle given distance from microtubule for the fitted model and the empirical distribution are shown for the median cell of each pattern (the same cells as shown in Fig 5 and S4 Fig). (TIF) [file pcbi.1004614.s001.tif]

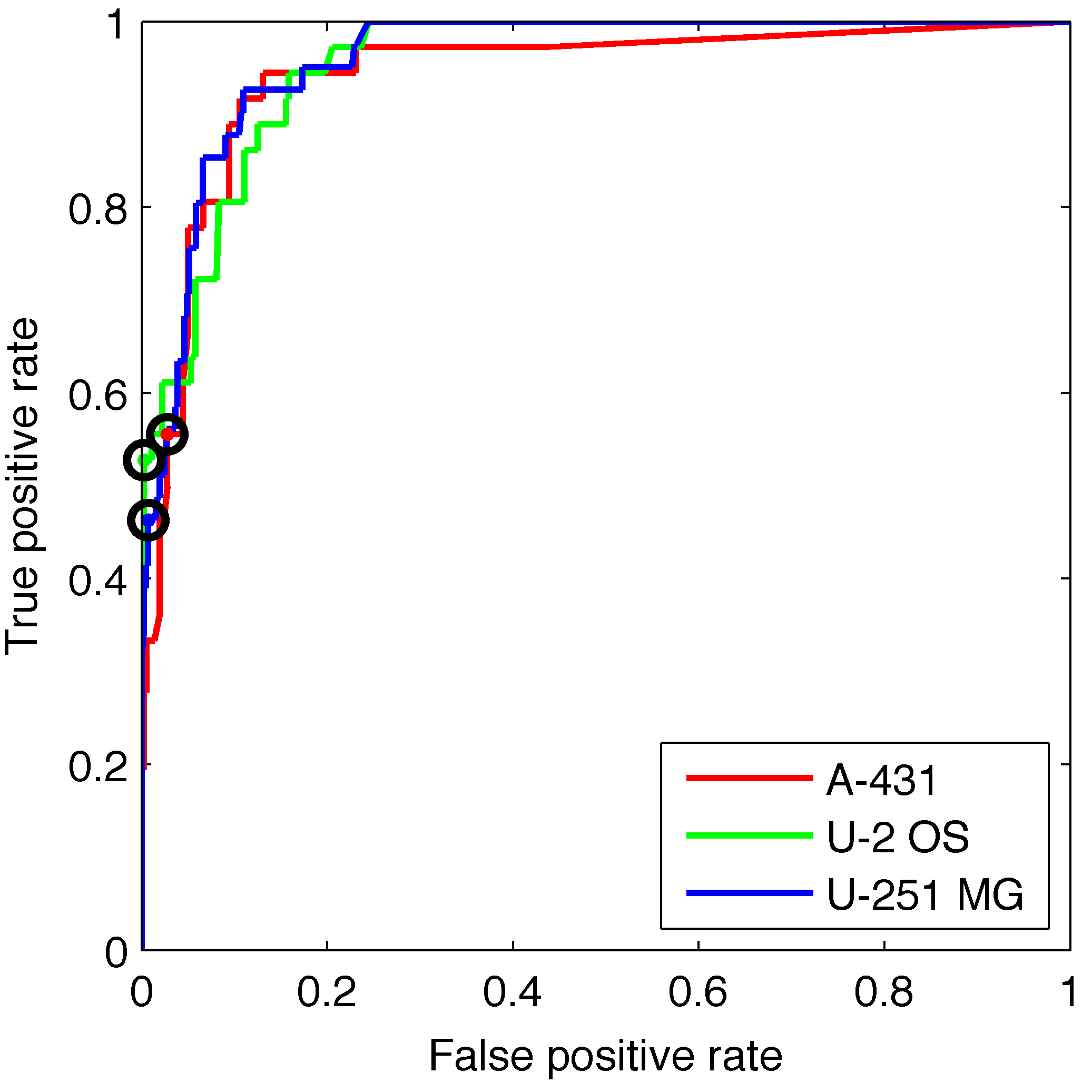

Supplement: S2 Fig — Receiver operating characteristic curves for the accuracy statistic for determining the in-class threshold are shown for the three cell types. The accuracy corresponding to the optimal threshold is shown as a black circle (see Methods). (TIF) [file pcbi.1004614.s002.tif]

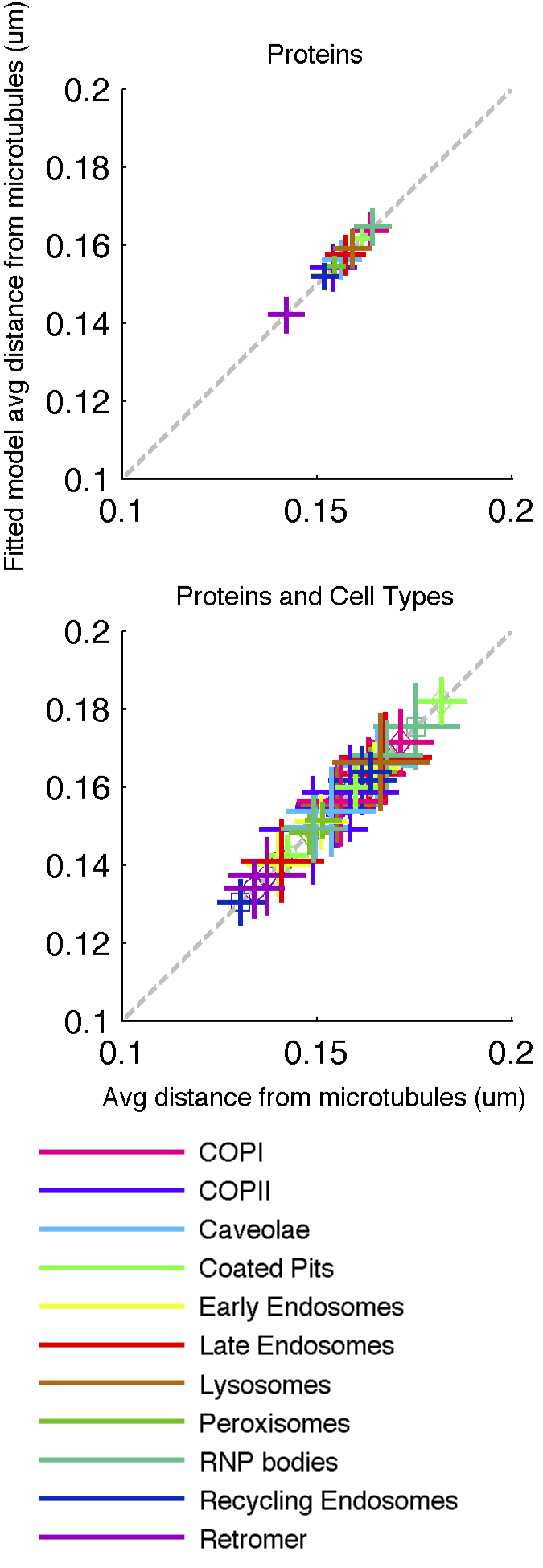

Supplement: S3 Fig — Each symbol represents a cell type; square for A-431, diamond for U-2 OS and circle for U-251 MG. The lines represent confidence intervals using Tukey’s range test for the empirical data (x-axis) and fitted model (y-axis) after 2-way ANOVA. (TIF) [file pcbi.1004614.s003.tif]

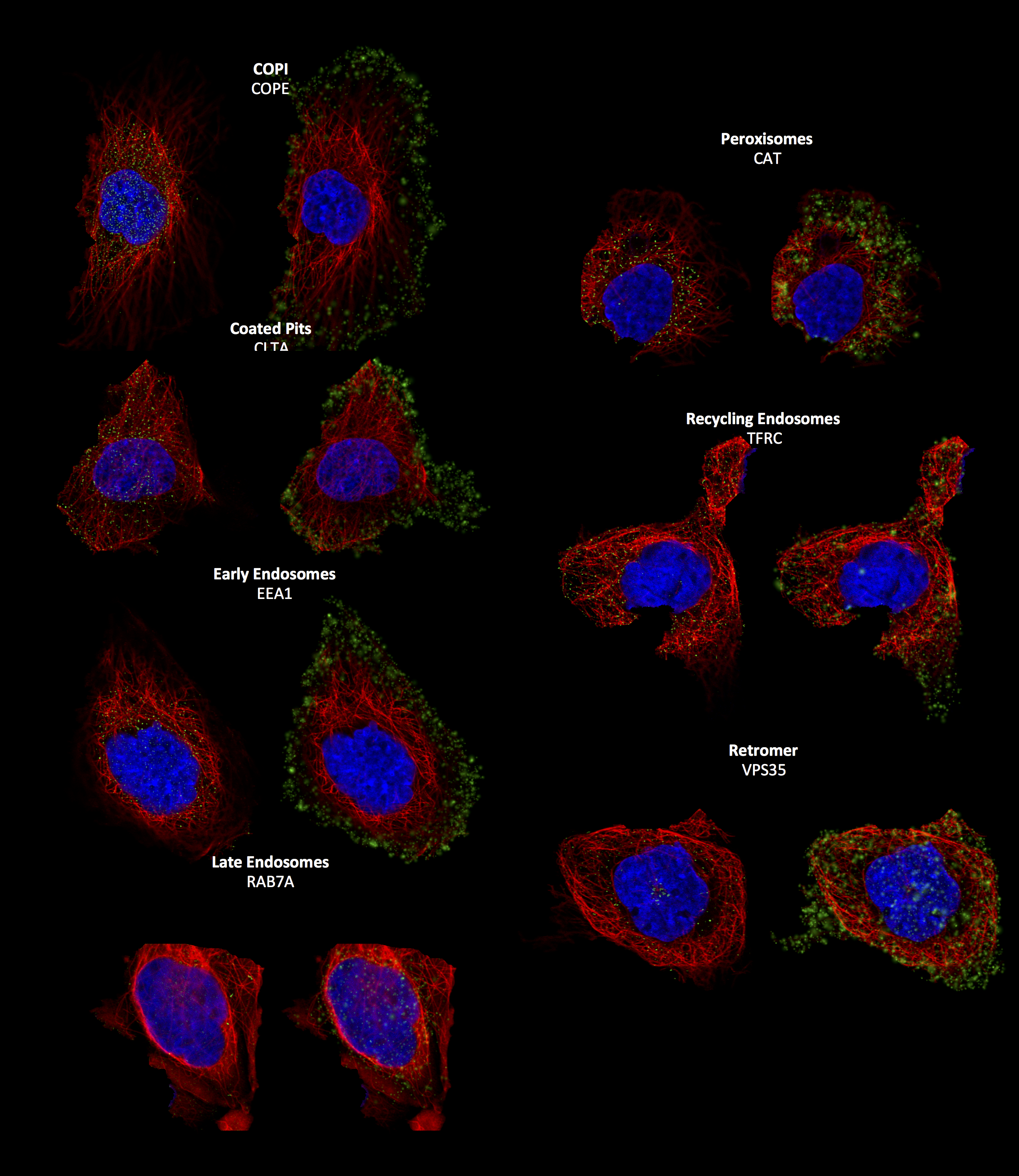

Supplement: S4 Fig — The left column shows cell images closest to the median of parameter space for cells of that pattern, and the right column shows synthesized protein patterns from the generative model of protein pattern conditional on cell geometry and microtubules of the left panel. (TIF) [file pcbi.1004614.s004.tif]
