# Supplementary material for: Automated Learning of Subcellular Variation among Punctate Protein Patterns and a Generative Model of Their Relation to Microtubules
Source: PLoS Comput Biol. 2015 Dec 1;11(12):e1004614. doi: 10.1371/journal.pcbi.1004614 (PMC4704559; doi:10.1371/journal.pcbi.1004614)
Supplement: S1 Table — Radial position is defined as r = L1/(L1+L2) where L1 is the distance between the center of each punctum and the nuclear membrane, and L2 is the distance from the center of each punctum to the cell membrane. Therefore, r is positive if the punctum is outside of the nucleus and negative inside. α is the angle between the major axis of the cell and the vector from the center of cell to the center of a punctum. The generative model component that a given feature is used for is also shown (see Fig 3). (DOCX) [file pcbi.1004614.s008.docx]

Table S1, Generative model parameters. Radial position is defined as *r*=*L*1/(*L*1+*L*2) where *L*1 is the distance between the center of each punctum and the nuclear membrane, and *L*2 is the distance from the center of each punctum to the cell membrane. Therefore, *r* is positive if the punctum is outside of the nucleus and negative inside. α is the angle between the major axis of the cell and the vector from the center of cell to the center of a punctum. The generative model component that a given feature is used for is also shown (see Figure 3).

| **Feature** | **Model Component** | **Description** |
| --- | --- | --- |
| mx1 | s_v_ | Average punctum size |
| mx2 | s_v_ | Average punctum intensity |
| mx3 | s_v_ | Size variance |
| mx4 | s_v_ | Size and intensity covariance |
| mx5 | s_v_ | Intensity variance |
| mx6 | n_v_ | Number of puncta |
| mx7 | p_p_ | Punctum position model β_0_ - intersect |
| mx8 | p_p_ | Punctum position model β_1_- radial position |
| mx9 | p_p_ | Punctum position model β_2_ - radial position^2^ |
| mx10 | p_p_ | Punctum position model β_3_ - angular pos. 1 sin(α) |
| mx11 | p_p_ | Punctum position model β_4_ - angular pos. 2 cos(α) |
| mx12 | p_p_ | Punctum position model β_5_ - distance from microtubules |
| mx13 | p_p_ | Punctum position model β_6_ - distance from microtubules^2^ |
| mx14 | p_b_ | Background position model β_0_ - intersect |
| mx15 | p_b_ | Background position model β_1_- radial position |
| mx16 | p_b_ | Background position model β_2_ - radial position^2^ |
| mx17 | p_b_ | Background position model β_3_ - angular pos. 1 sin(α) |
| mx18 | p_b_ | Background position model β_4_ - angular pos. 2 cos(α) |
| mx19 | p_b_ | Background position model β_5_ - distance from microtubules |
| mx20 | p_b_ | Background position model β_6_ - distance from microtubules^2^ |
| mx21 | N/A | Total intensity of puncta |
| mx22 | i_b_ | Total intensity of background |
